# Effects of *NFKB1* and *NFKBIA* Gene Polymorphisms on Hepatocellular Carcinoma Susceptibility and Clinicopathological Features

**DOI:** 10.1371/journal.pone.0056130

**Published:** 2013-02-14

**Authors:** Chao-Wen Cheng, Jen-Liang Su, Chiao-Wen Lin, Chun-Wen Su, Chun-Han Shih, Shun-Fa Yang, Ming-Hsien Chien

**Affiliations:** 1 Graduate Institute of Clinical Medicine, College of Medicine, Taipei Medical University, Taipei, Taiwan; 2 Graduate Institute of Cancer Biology, College of Medicine, China Medical University, Taichung, Taiwan; 3 Center for Molecular Medicine, China Medical University Hospital, Taichung, Taiwan; 4 Department of Biotechnology, Asia University, Taichung, Taiwan; 5 Graduate Institute of Toxicology, College of Medicine, National Taiwan University, Taipei, Taiwan; 6 Institute of Oral Sciences, Chung Shan Medical University, Taichung, Taiwan; 7 Institute of Medicine, Chung Shan Medical University, Taichung, Taiwan; 8 Department of Trauma and Emergency Surgery, Wan Fang Hospital, Taipei Medical University, Taipei, Taiwan; 9 Department of Medical Research, Chung Shan Medical University Hospital, Taichung, Taiwan; 10 Wan Fan Hospital, Taipei Medical University, Taipei, Taiwan; University of Illinois at Chicago, United States of America

## Abstract

**Background:**

Constitutive activation of nuclear factor (NF)-κB is frequently observed in hepatocellular carcinoma (HCC). The current study examined associations of polymorphisms within promoter regions of *NFKB1* encoding NF-κB1 and *NFKBIA* encoding IκBα with the susceptibility of developing HCC and clinicopathological characteristics of the tumors.

**Methodology and Principal Findings:**

Genetic polymorphisms of *NFKB1* and *NFKBIA* were analyzed by a real-time polymerase chain reaction (PCR) in 135 HCC patients and 520 healthy controls. The genotypic frequency of the *NFKB1* -94 Ins polymorphism in HCC patients was significantly higher than that of the controls (adjusted odds ratio (AOR) = 2.23; 95% confidence interval (CI) 1.32∼3.77). No statistical significance was observed for the distribution frequency of the *NFKBIA* −-519 C/T, -826 C/T, or -881 A/G genotype and haplotype polymorphisms between HCC patients and controls. Furthermore, female HCC patients carrying the *NFKB1* -94 Ins polymorphism were associated with lower clinical stages and smaller tumor sizes.

**Conclusions:**

Our results indicate that the *NFKB1* -94 Ins promoter polymorphism increased the risk of HCC, and may be applied as a predictive factor for the clinical stage and tumor size in female HCC patients.

## Introduction

Hepatocellular carcinoma (HCC) is the most common primary liver cancer and the second leading cause of cancer-related deaths in Taiwan; thus, HCC is one of the most important cancers worthy of our concern [1]. Epidemiologic features include differences in geographic regions, gender disparities, racial and ethnic groups, and exposure to certain environmental factors [2]. Chronic hepatitis B virus (HBV) and hepatitis C virus (HCV) infection, cirrhosis, carcinogen exposure, excessive alcohol consumption, and genetic factors are considered multiple risk factors that contribute to hepatocarcinogenesis [3]–[6]. Several single-nucleotide polymorphisms (SNPs) were identified in genes encoding for stromal cell-derived factor (SDF)-1, E-cadherin, and tumor necrosis factor (TNF)-α and are predictable risk factors for HCC [7]–[9]. Investigating differential inherited genetic alternations may contribute to an understanding of hepatocarcinogenesis and can be further applied for preventative interventions.

Nuclear factor (NF)-κB is an important transcription factor for maintaining normal immune system function; inadequate NF-κB activation can mediate inflammation and tumorigenesis [10], [11]. In an unstimulated condition, NF-κB is sequestrated in the cytoplasm and inhibits transcriptional activation by binding to inhibitors of NF-κB (IκB). Once IκB proteins are phosphorylated and degraded, NF-κB is subsequently released and further translocated into nuclei, where gene transcription is initiated [12]. Activation of NF-κB is rarely observed in normal cells except for proliferating T cells, B cells, thymocytes, monocytes, and astrocytes, while it is constitutively active in most tumor cells [13], [14]. In HCC, hepatic expression of NF-κB is also constitutively activated in HCC tissue samples compared to surrounding liver tissues [15]–[17].

Functional nucleotide polymorphisms in either *NFKB1* or its inhibitory protein, IκB, can potentially regulate NF-κB signaling and contribute to the carcinogenesis of HCC. Among the five members of the NF-κB family (p50, p65/Rel A, c-Rel, Rel B, and p52) in mammalian cells, the major form of NF-κB is a heterodimer of the p50 and p65/Rel A subunits [18], [19]. The p50 subunit, encoded by the *NFKB1* gene located on chromosome 4q23–24, has a common -94 Del/Ins polymorphism in the promoter region. The promoter sequence containing the -94 Ins polymorphism increases *NFKB1* messenger (m)RNA expression and is associated with susceptibility to ulcerative colitis [20]. IκBα encoded by the *NFKBIA* gene comprises a relatively large number of polymorphisms. *NFKBIA* -519 C/T, -826 C/T, and -881 A/G polymorphisms are respectively located at putative binding sites for transcription factors CCAAT/enhancer binding protein, GATA binding protein 2, and retinoic acid-related orphan receptor α, [21], [22] may regulate IκBα expression, and hence influence NF-κB activation. Recent studies showed genetic polymorphisms of the *NFKB1* and *NFKBIA* genes to be associated with cancer risk and severity in sporadic colorectal cancer and oral cancer, [23], [24] but their possible associations with predictions of risk and prognosis of HCC remain poorly investigated. In this study, we attempted to determine the importance of *NFKB1* and *NFKBIA* gene promoter polymorphisms to the occurrence of HCC in Taiwanese and evaluated their relevance by correlating them with tumor clinicopathological characteristics.

## Materials and Methods

### Subjects and Specimen Collection

The present hospital-based case-control study recruited 135 HCC patients in 2007∼2010 at Chung Shan Medical University Hospital, Taichung, Taiwan. A diagnosis of HCC was based on characteristic criteria of national guidelines for HCC, such as liver tumor tissue diagnosed by histology or cytology irrespective of the α-fetoprotein (AFP) titer where imaging data, either computed tomography or magnetic resonance imaging, showed one of following: (1) one or more liver masses of ≥2 cm in diameter; (2) imaging data with early enhancement and a high level of AFP of ≥400 ng/mL; and (3) imaging data with early arterial phase-contrast enhancement plus early venous phase-contrast washout. During the same study period, 520 race- and ethnic group-matched individuals were enrolled as the controls who entered the physical examination at the same hospital. These control groups had neither self reported history of cancer of any sites. Personal information and characteristics collected from the study subjects using interviewer-administered questionnaires contained questions involving demographic characteristics and the status of cigarette smoking and alcohol drinking. Nonsmokers were defined as individuals who had never smoked or had smoked for less than one year, and others were defined as smokers. Non-drinkers were defined as those who had never drank or had drank less than once per week and/or for less than one year, and others were defined as alcohol drinkers.

HCC patients were clinically staged at the time of diagnosis according to the TNM staging system of the *American Joint Committee on Cancer (AJCC) Staging Manual* (7th ed.) [24]. Liver cirrhosis was diagnosed with a liver biopsy, abdominal sonography, or biochemical evidence of liver parenchymal damage with endoscopic esophageal or gastric varices. The patients’ clinicopathological characteristics, such as clinical stage, tumor size, lymph-node metastasis, distant metastasis, hepatitis B surface antigen (HBsAg), antibody to HCV (anti-HCV), liver cirrhosis, AFP, aspartate aminotransferase (AST), and alanine aminotransferase (ALT), were verified by a chart review. Whole-blood specimens collected from healthy controls and HCC patients were placed in tubes containing ethylenediaminetetraacetic acid (EDTA), immediately centrifuged, and stored at −80°C. Before conducting the study, approval from the Institutional Review Board of Chung Shan Medical University Hospital was obtained, and informed written consent was obtained from each individual.

### Genomic DNA Extraction

Genomic DNA was extracted using QIAamp DNA blood mini kits (Qiagen, Valencia, CA, USA) following the manufacturer’s instructions. We dissolved DNA in TE buffer (10 mM Tris at pH 7.8 and 1 mM EDTA) and then quantified it by measuring the optical density at 260 nm. The final preparation was stored at −20°C and used to create templates for the polymerase chain reaction (PCR).

### Real-time PCR

Allelic discrimination of the *NFKB1* -94, *NFKBIA* -519, *NFKBIA* -826, and *NFKBIA* -881 gene polymorphisms were assessed with the ABI StepOne™ Real-Time PCR System (Applied Biosystems, Foster City, CA, USA) and analyzed using SDS v3.0 software (Applied Biosystems), with the TaqMan assay [25]. The final volume for each reaction was 5 µL, containing 2.5 µL TaqMan Genotyping Master Mix, 0.125 µL TaqMan probe mix, and 10 ng genomic DNA. The real-time PCR included an initial denaturation step at 95°C for 10 min, followed by 40 cycles of 95°C for 15 s and 60°C for 1 min.

### Statistical Analyses

Differences between groups were considered significant for *p* values of <0.05. Hardy-Weinberg equilibrium (HWE) was assessed using a goodness-of-fit *Χ* ^2^-test for biallelic markers. The Mann-Whitney U-test and Fisher’s exact test were used to compare differences in demographic characteristic distributions between the healthy control group and HCC patients. The adjusted odds ratios (AORs) and 95% confidence intervals (CIs) of the association of genotype frequencies with risk and clinicopathological characteristics were estimated using multiple logistic regression models after controlling for other covariates. We analyzed all data with Statistical Analytic System (SAS Institute, Cary, NC, USA) software (vers. 9.1, 2005) for Windows.

## Results

In this case-control study, the statistical analysis of demographic characteristics of HCC patients showed significant differences in the distribution of ages and genders compared to the controls, but not in alcohol or tobacco consumption (Table 1). To reduce the possible interference of confounding variables, we used AORs with 95% CIs estimated by multiple logistic regression models after controlling for age and gender in each comparison.

**Table 1:** Distributions of demographic characteristics in the 520 controls and 135 patients with hepatocellular carcinoma.

Variable	Controls (*N* = 520)	Patients (*N* = 135)	*p* value
Age (yr)	Mean ± S.D.	Mean ± S.D.	
	52.43±14.67	64.24±11.08	<0.001*
**Gender**	***n*** ** (%)**	***n*** ** (%)**	
Male	426 (81.9%)	92 (68.1%)	
Female	94 (18.1%)	43 (31.9%)	<0.001*
**Alcohol consumption**			
No	309 (59.4%)	88 (65.2%)	
Yes	211 (40.3%)	47 (34.8%)	= 0.222
**Tobacco consumption**			
No	310 (59.6%)	80 (59.3%)	
Yes	210 (40.4%)	55 (40.7%)	= 0.940
**Stage**			
I		53 (39.3%)	
II		31 (23.0%)	
III		44 (32.6%)	
IV		7 (5.2%)	
**Tumor T status**			
≤T2		87 (64.4%)	
>T2		48 (35.6%)	
**Lymph node status**			
N0		128 (94.8%)	
N1+N2		7 (5.2%)	
**Metastasis**			
M0		129 (95.6%)	
M1		6 (4.4%)	

Mann-Whitney U test or Fisher’s exact test was used between healthy controls and patients with HCC.

* *p*<0.05 which was statistically significant.

Genotype distributions of promoter polymorphisms in *NFKB1* -94 Del/Ins and *NFKBIA* -519 C/T, -826 C/T, and -881 A/G between patients and controls are presented in Table 2. For the control group, all analyzed gene markers were in HWE (*p*>0.05). After adjusting for other confounders, a significantly increased risk was found for HCC patients carrying the *NFKB1* -94 Ins polymorphism compared to those homozygous for the -94 Del polymorphism. Compared to corresponding wild-type (WT) homozygotes of the control group, the risk for HCC was 1.85-fold (95% CI: 1.04∼3.27) for -94 Del/Ins, 3.52-fold (95% CI: 1.75∼7.10) for -94 Ins/Ins, and 2.23-fold (95% CI: 1.32∼3.77) higher for the combination of -94 Del/Ins and Ins/Ins. However, the -519 C/T, -826 C/T, and -881 A/G gene polymorphisms of the *NFKBIA* gene showed no significant differences in HCC risk for individuals with NFKBIA -519, -826, or -881 polymorphic genes compared to those with the WT gene. We further explored haplotypes to evaluate the combined effect of the three *NFKBIA* polymorphisms on HCC susceptibility. Distribution frequencies of *NFKBIA* -519 C/T, -826 C/T, and -881 A/G haplotypes in our recruited individuals were analyzed. There were three haplotypes with frequencies of >5% among all cases; the most common haplotype in the control was CCA (83.1%), and it was therefore chosen as a reference. After comparison to the reference, haplotypes of -519, -826, and -881 combined did not predict susceptibility to HCC either (Table 3).

**Table 2:** Adjusted odds ratio (OR; AOR) and 95% confidence interval (CI) for hepatocellular carcinoma (HCC) associated with nuclear factor (NF)-κB and inhibitor of NF-κB (IκB) genotypic frequencies.

Variable	Controls (*N* = 520) *n* (%)	Patients (*N* = 135) *n* (%)	OR (95% CI)	AOR (95% CI)
**NF-κB**				
Del/Del	168 (32.3%)	29 (21.5%)	1.00	1.00
Del/Ins	271 (52.1%)	64 (47.4%)	1.37 (0.85∼2.21)	1.85 (1.05∼3.27)*
Ins/Ins	81 (15.6%)	42 (31.1%)	3.00 (1.75∼5.17)*	3.52 (1.75∼7.10)*
Del/Ins+ Ins/Ins	352 (67.7%)	106 (78.5%)	1.75 (1.11∼2.74)*	2.23 (1.32∼3.77)*
**IκB -519**				
CC	432 (83.1%)	117 (86.7%)	1.00	1.00
CT	86 (16.5%)	17 (12.6%)	0.73 (0.42∼1.28)	0.68 (0.35∼1.29)
TT	2 (0.4%)	1 (0.7%)	1.85 (0.17∼20.54)	3.68 (0.26∼52.95)
CT+TT	88 (16.9%)	18 (13.3%)	0.76 (0.44∼1.31)	0.72 (0.38∼1.35)
**IκB -826**				
CC	438 (84.2%)	106 (78.5%)	1.00	1.00
CT	78 (15.0%)	27 (20.0%)	1.43 (0.88∼2.33)	1.44 (0.80∼2.62)
TT	4 (0.8%)	2 (1.5%)	2.07 (0.37∼11.43)	1.54 (0.14∼16.51)
CT+TT	82 (15.8%)	29 (21.5%)	1.46 (0.91∼2.35)	1.44 (0.80∼2.57)
**IκB -881**				
AA	438 (84.2%)	106 (78.5%)	1.00	1.00
AG	78 (15.0%)	27 (20.0%)	1.43 (0.88∼2.33)	1.44 (0.80∼2.62)
GG	4 (0.8%)	2 (1.5%)	2.07 (0.37∼11.43)	1.54 (0.14∼16.51)
AG+GG	82 (15.8%)	29 (21.5%)	1.46 (0.91∼2.35)	1.44 (0.80∼2.57)

The ORs with their 95% CIs were estimated by a logistic regression model. The AORs with their 95% CIs were estimated by a multiple logistic regression model after controlling for age, gender, and alcohol and tobacco consumption.

**Table 3:** Distribution frequency of the *IκB* haplotype in controls and hepatocellular carcinoma patients.

Variable			Controls (*N* = 1040)*n* (%)	Patients (*N* = 270)*n* (%)	OR (95% CI)	*p* value	
-519 C/T	-826 C/T	-881 A/G					
C	C	A	864 (83.1%)	221 (81.9%)	Reference		
T	C	A	90 (8.7%)	18 (6.6%)	0.78 (0.46∼1.33)		0.359
C	T	G	86 (8.3%)	30 (11.1%)	1.36 (0.88∼2.12)		0.167
T	T	G	0 (0%)	1 (0.4%)	–		

OR, odds ratio; CI confidence interval.

This study also analyzed relationships of the clinical status with the *NFKB1* -94 Del/Ins polymorphism and *NFKBIA* -519 C/T, -826 C/T, and -881 A/G polymorphisms. The status of the *NFKBIA* -519 C/T, -826 C/T, and -881 A/G polymorphisms did not show significant associations with any clinicopathological characteristics (data not shown). In turn, *NFKB1* -94 Del/Ins promoter polymorphisms presented significant differences with clinical stage (AOR: 0.218; 95% CI: 0.056∼0.985) (*p*<0.05) and tumor size (AOR: 0.200; 95% CI: 0.041∼0.981) (*p*<0.05) in female HCC patients with at least one *NFKB1* -94 Ins polymorphism, while no associations were found in any male HCC patients. A higher proportion of females carrying the *NFKB1* -94 Ins polymorphism were in stage I/II than in stage III/IV (84.4% vs. 15.6%, respectively); tumor sizes also presented similar results (≤T2 vs. >T2; 87.5% vs. 12.5%, respectively) (Table 4).

**Table 4:** Clinical status and *NF-κB* genotypic frequencies in 135 hepatocellular carcinoma (HCC) patients.

Variable	NF-κB (All cases)			NF-κB (Male)			NF-κB (Female)		
	Del/Del (*n* = 29)	Del/Ins+ Ins/Ins (*n* = 106)	AOR (95% CI)^a^	Del/Del (*n* = 18 )	Del/Ins+ Ins/Ins (*n* = 74)	AOR (95% CI)^b^	Del/Del (*n* = 11 )	Del/Ins+ Ins/Ins (*n* = 32)	AOR (95% CI)^b^
**Clinical Stage**									
Stage I/II	15 (51.7%)	69 (65.1%)	Reference	9 (50.0%)	42 (56.8%)	Reference	6 (54.5%)	27 (84.4%)	Reference
Stage III/IV	14 (48.3%)	37 (34.8%)	0.536 (0.154–1.861)	9 (50.0%)	32 (43.2%)	0.451 (0.073–2.786)	5 (45.5%)	5 (15.6%)	0.218 (0.056–0.985)*
**Tumor size**									
≤T2	16 (55.2%)	71 (67.0%)	Reference	10 (55.6%)	43 (58.1%)	Reference	6 (54.5%)	28 (87.5%)	Reference
>T2	13 (44.8%)	35 (33.0%)	0.573 (0.166–1.975)	8 (44.4%)	31 (41.9%)	0.688 (0.124–3.809)	5 (45.5%)	4 (12.5%)	0.200 (0.041–0.981)*
**Lymph node metastasis**									
No	27 (93.1%)	101 (95.3%)	Reference	16 (88.9%)	70 (94.6%)	Reference	11 (100%)	31 (96.9%)	Reference
Yes	2 (6.9%)	5 (4.7%)	0.351 (0.029–4.236)	2 (11.1%)	4 (5.4%)	0.136 (0.005–3.383)	0 (0%)	1 (3.1%)	–
**Distant metastasis**									
No	28 (96.6%)	101 (95.3%)	Reference	17 (94.4%)	70 (94.6%)	Reference	11 (100%)	31 (96.9%)	Reference
Yes	1 (3.4%)	5 (4.7%)	1.026 (0.060–17.488)	1 (5.6%)	4 (5.4%)	0.356 (0.010–12.727)	0 (0%)	1 (3.1%)	–

>T2: multiple tumor of >5 cm or tumor involving a major branch of the portal or hepatic veins.

a The AORs with their 95% CIs were estimated by multiple logistic regression models after controlling for age, gender, smoking and drinking.

b The AORs with their 95% CIs were estimated by multiple logistic regression models after controlling for age, gender, smoking and drinking.

* *P* value <0.05 as statistically significant.

Relationships between genotypic frequencies and levels of hepatic clinicopathological laboratory indicators, such as AFP, AST, ALT, and the ratio of AST to ALT in HCC patients, were evaluated. Results showed that hepatic clinicopathological laboratory indicators and the genotypic distributions of the *NFKB1* and *NFKBIA* genes did not exhibit significant associations (Table 5).

**Table 5:** Association of the nuclear factor (NF)-κB and inhibitor of NF-κB (IκB) genotypic frequencies with the hepatocellular carcinoma laboratory status.

Characteristic	α-Fetoprotein (ng/mL)	AST (IU/L)	ALT (IU/L)	AST/ALT ratio
**NF-κB**				
Del/Del	2043.0±1217.6	130.4±33.9	102.4±22.3	1.72±0.33
Del/Ins+ Ins/Ins	3775.0±1631.7	194.4±33.4	153.9±28.0	1.44±0.08
* p* value	0.588	0.388	0.350	0.238
**IκB -519**				
CC	3907.4±1503.5	185.2±34.7	148.0±25.9	1.46±0.10
CT/TT	123.7±44.7	151.2±28.1	109.4±17.6	1.81±0.33
* P* value	0.327	0.704	0.563	0.215
**IκB -826**				
CC	3619.6±1625.0	196.0±37.6	148.4±27.4	1.53±0.11
CT/TT	2610.9±1355.0	124.6±30.3	122.7±32.4	1.40±0.18
* P* value	0.753	0.335	0.642	0.582
**IκB -881**				
AA	3619.6±1625.0	196.0±37.6	148.4±27.4	1.53±0.11
AG/GG	2610.9±1355.0	124.6±30.3	122.7±32.4	1.40±0.18
* P* value	0.753	0.335	0.642	0.582

## Discussion

The common insertion/deletion promoter polymorphism, -94 Del/Ins, in the *NFKB1* gene was reported to have regulatory ability over *NFKB1* gene expression. It is located between two putative key promoter regulatory elements, and the promoter sequence containing the -94 Ins polymorphism had higher activities than a comparable sequence containing the -94 Del polymorphism. Oligonucleotides that contained with -94 Del sequence in the *NFKB1* promoter had reduced nuclear protein-binding ability; in addition, the -94 Del *NFKB1* promoter also showed decreased promoter activity [20]. This evidence suggests that the *NFKB1* promoter with the -94 Ins polymorphism may affect the NF-κB expression level through enhancing the transcription factor-binding ability and promoter activity.

A recent meta-analysis study also indicated an association between the *NFKB1* -94 Ins promoter polymorphism and the incidence of cancer in Caucasian and Asian populations. [23] Thus, the -94 Ins promoter polymorphism increasing the risk of having cancer may result from its positive regulation of NF-κB expression. In addition, carriers of the -94 Ins polymorphism also presented increased susceptibility to prostate cancer, gastric cancer, nasopharyngeal carcinoma, and oral squamous cell carcinoma, [24], [26]–[28] although this was not supported by sporadic breast cancer in a Caucasian population [29]. In a study on HBV-induced hepatocarcinogenesis, the *NFKB1* -94 Ins polymorphism also showed a greater prevalence in HCC patients than in healthy controls [30]. In our study (Table 2), the frequency of the -94 Ins polymorphism in Taiwanese HCC patients was significantly higher than that in control subjects, indicating that the *NFKB1* -94 Ins promoter polymorphism increased the incidence of HCC in Taiwanese. HCC patients bearing the *NFKB1* -94 Ins polymorphism may have higher NF-κB expression levels and be sensitized to abnormal responses to carcinogenic stimuli, leading to its constitutive activation and tumorigenesis.

The NFKBIA (IκBα) protein is the main regulator of NF-κB activation through conjugation with the NF-κB protein in cytoplasmic sequestration and inhibition of its transcriptional activation [31]. The differential expression level of IκBα may influence activation of NF-κB transactivated genes [32]; therefore, promoter polymorphisms of the *NFKBIA* polymorphism may present another regulatory mechanism modulating NF-κB-mediated HCC carcinogenesis. Three polymorphisms in the promoter region of *NFKBIA* were selected (-519 C/T, -826 C/T, and -881 C/T) to study the association with HCC. In the in vitro *NFKBIA* promoter luciferase assay, constructs with the -826C, -550A, -519T, -826T, -550A, and -519T genotypes were expressed at one-half the activity level of other polymorphic constructs [22], suggesting that the -519T polymorphism may have lower IκBα protein expression. Carrying the *NFKBIA* -519T polymorphism was reported to be inversely associated with gastric and liver cancer [10], [30]. Carrying the -881G and -826T polymorphisms may also mediate increased NF-κB expression in patients with sarcoidosis [11], [33]. In this study, promoter polymorphisms and the distribution frequencies of haplotype polymorphisms of *NFKBIA* -519 C/T, -826 C/T, and -881 A/G presented no significant differences in terms of the association with the risk of HCC in Taiwanese patients. It was reported that carrying the *NFKBIA* -881G and -826T polymorphisms was associated with higher risks of HCC compared to HBV-infected non-HCC subjects, but not in healthy controls [30]. Those results suggest that these two promoter polymorphisms may be linked to HBV-induced hepatocarcinogenesis, but the linkage for normal individuals requires further investigation.

The -94 Ins polymorphism in *NFKB1* promoter has been suggested to enhance the activity and increased the production of transcription factor NF-κB, thus, the hepatocytes may present with higher susceptibility to carcinogenic stimuli and leading to HCC. The main causes of HCC in Taiwan are chronic hepatitis B and C infections, higher than 90% of patients positive for hepatitis B surface antigen (HBsAg) or antibody to hepatitis C virus (anti-HCV) [34]. In addition, the viral gene products of HBV or HCV had presented the abilities in regulating NF-κB, AP-1, and STAT3 activation [35]–[37]. Higher NF-κB gene expression in individuals with -94 Ins *NFKB1* promoter may increase the susceptibility to viral gene products mediated activation and the risk of HCC. This inference was supported by our observations where patients with -94 Ins *NFKB1* promoter polymorphism showed a higher incidence of HCC (Table 2). By analyzing the clinical status and frequency of the *NFKB1* -94 Del/Ins polymorphism in 135 HCC patients, the parameters of clinical stage, tumor size, lymph node metastasis, and distant metastasis presented no significant differences in any HCC case. The incidence of HCC in men was >2-fold higher than that in women in almost all populations [2]. The 135 cases in this study, including 68.1% males and 31.9% females, also presented a ratio of 2.13∶1 (Tables 1, 4). In terms of gender differences, clinical stages and tumor sizes showed significant differences between the -94 Ins and -94 Del polymorphisms in female carriers, but not in male carriers. Female carriers with -94 Ins showed a higher proportion in stage I/II than with -94 Del (84.4% vs. 54.5%); tumor sizes of T2 also presented similar results (87.5% vs. 54.5%). The -94 Ins polymorphism in the *NFKB1* promoter was inversely associated with the clinical stage and tumor size in female patients, which suggests that certain factors in relationship with gender disparity may influence the malignant pathological features of HCC by manipulating the NF-κB-mediated signal pathway.

The risk of HCC in females was reported inversely correlated to the age at natural menopause and to the number of full-term pregnancies. Oophorectomy at age <50 during premenopausal years was a risk factor, in turn, postmenopausal hormone replacement therapy was associated with a lower risk of HCC [38]. This information implies that the activity of estrogens in female patients may regulate the pathogenesis of HCC. In an animal study of chemically induced HCC, diethylnitrosamine caused HCC in 100% of male mice but only in 10%∼30% of female littermates. The difference relates to estrogen's inhibition of NF-κB mediated IL-6 production by Kupffer cells [39]. Estrogen can also modulate HCC malignancy in vivo by reducing tumor cell invasion, arresting cell-cycle progression, and promoting apoptosis through NF-κB inhibition [40]. In addition to HCC, the NF-κB-binding activity also presented a significant inverse relationship with the ER content in ER-positive breast tumors [21]. The interaction between estrogen and NF-κB activities could in part explain the inverse correlation between -94 Ins polymorphism in the *NFKB1* promoter and the clinical stage and tumor size in female patients. The female HCC patients carrying the *NFKB1* -94 Del polymorphism may exhibit less sensitization to ER activation and estrogen-based therapy may be more effective in patients carrying the *NFKB1* -94 Ins promoter, but less effective in *NFKB1* -94 Del patients. Therefore, treatment with estrogen may suppress NF-κB-mediated hepatocarcinogenesis and this concept can be further applied for personalized HCC therapeutic approaches.

In Taiwan, HBsAg was positive in approximately 80% of male patients with HCC in 1981–1983, gradually decreased to 68% in 1996–1998 and to 66% in 1999–2001. It is also of note that HCV has replaced HBV in the viral etiology of HCC gradually, and has become the main etiology of HCC [34]. In this case control study (patients recruited from 2007 to 2010), 40% of all HCC patients were positive for HBsAg and 53.3% were positive in anti-HCV antibody. The polymorphism of *NFKB1* may affect the susceptibility to these viral infections-related malignancies; however, due to the lack of patients’ detail history of infections, the results in this study may be limited. A global neonatal vaccination program against HBV was launched in 1984 in Taiwan; the HBsAg carrier rate had decreased from the historical 15–20% to <1% after vaccination. This achievement may also help to delineate the association between *NFKB1* polymorphism and HBV in the pathogenesis of HCC by further studies in younger generation patients. Another limitations of our study is that information regarding HCC risk factors such as alcohol and tobacco consumption was presented as “ever” versus “never.” As such, we were unable to perform a more detailed analysis by stratifying individuals on the basis of amount, length, and history of alcohol drinking or cigarette smoking. In addition, the number of HCC cases was small, which limited the statistical power to detect small effects. Future studies with larger sample sizes could provide additional support to our findings in this study. Furthermore, the functional role of *NFKB1* -94 Ins promoter polymorphism in growth or metastasis of HCC is worth for further investigation, which will be included in our future work. Clones containing various genotypes of *NFKB1* -94 SNPs will be constructed to elucidate the possible functions of NF-κB (proliferation and cell cycle regulation) in HCC cell lines, as well as the underlying mechanisms.

In conclusion, we showed that the *NFKB1* -94 Ins promoter polymorphism appeared to increase the risk of developing HCC. Female HCC patients who carried the -94 Ins polymorphism were associated with a lower clinical stage and a smaller tumor size. The 4-bp promoter polymorphism, -94 Del/Ins, in *NFKB1* promoter variants may represent a predisposing factor for HCC susceptibility in the Taiwanese population.
